# Assessing the Effect of Loop Mutations in the Folding Space of β_2_-Microglobulin with Molecular Dynamics Simulations

**DOI:** 10.3390/ijms140917256

**Published:** 2013-08-22

**Authors:** Sílvia G. Estácio, Eugene I. Shakhnovich, Patrícia F. N. Faísca

**Affiliations:** 1 Centre for Condensed Matter Physics, University of Lisbon, Av. Prof. Gama Pinto 2, Lisboa 1649-003, Portugal; 2 Department of Physics, University of Lisbon, Av. Prof. Gama Pinto 2, Lisboa 1649-003, Portugal; 3 Department of Chemistry and Chemical Biology, Harvard University, 12 Oxford Street, Cambridge, MA 02138, USA

**Keywords:** intermediate states, molten globule, folding pathways, discrete molecular dynamics, principal component analysis, dialysis-related amyloidosis

## Abstract

We use molecular dynamics simulations of a full atomistic Gō model to explore the impact of selected DE-loop mutations (D59P and W60C) on the folding space of protein human β_2_-microglobulin (Hβ_2_m), the causing agent of dialysis-related amyloidosis, a conformational disorder characterized by the deposition of insoluble amyloid fibrils in the osteoarticular system. Our simulations replicate the effect of mutations on the thermal stability that is observed in experiments *in vitro*. Furthermore, they predict the population of a partially folded state, with 60% of native internal free energy, which is akin to a molten globule. In the intermediate state, the solvent accessible surface area increases up to 40 times relative to the native state in 38% of the hydrophobic core residues, indicating that the identified species has aggregation potential. The intermediate state preserves the disulfide bond established between residue Cys25 and residue Cys80, which helps maintain the integrity of the core region, and is characterized by having two unstructured termini. The movements of the termini dominate the essential modes of the intermediate state, and exhibit the largest displacements in the D59P mutant, which is the most aggregation prone variant. PROPKA predictions of p*K*_a_ suggest that the population of the intermediate state may be enhanced at acidic pH explaining the larger amyloidogenic potential observed *in vitro* at low pH for the WT protein and mutant forms.

## 1. Introduction

**Figure 1:**
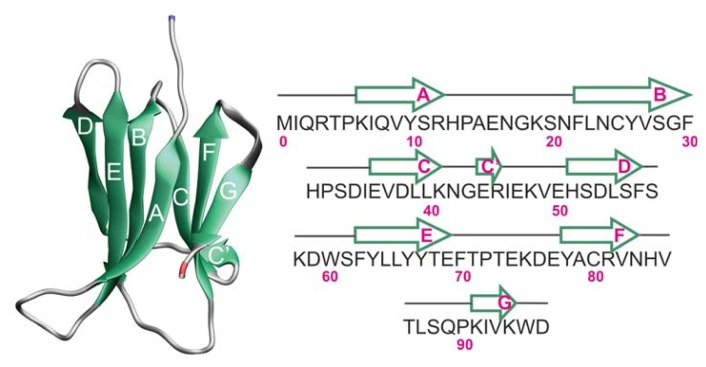
The native structure of wild-type (WT) human beta-2 microglobulin (Hβ_2_m) (PDB ID: 1LDS) and its primary sequence. The location of each β-strand along the protein sequence is also shown. In the single-point mutants, the secondary structure assignment is identical. Hβ_2_m exhibits a sandwich-like structure formed by two sheets of anti-parallel β-strands. One of the sheets comprises strands A-B-E-D with the second sheet being formed by strands C-F-G. The native structure is stabilized by a disulfide bond established between Cys25 (on strand B) and Cys80 (on strand F). Another key structural feature is the existence of a peptidyl-prolyl bond between His31 and Pro32 (on the BC-loop) which adopts a thermodynamically unfavorable *cis* isomerization in the native structure.

Human β_2_-microglobulin (Hβ_2_m) is the non-covalently bound light chain of the human class I major histocompatibility complex (MHC-I), where it chaperones the complex assembly for antigen presentation. The wild-type (WT) Hβ_2_m comprises 99 residues arranged into a typical immunoglobulin (Ig) fold. It exhibits a sandwich-like structure formed by two sheets of antiparallel β-strands. One of the sheets comprises strands A-B-E-D with the second sheet being formed by strands C-F-G. The native structure is stabilized by a disulfide bond established between residue Cys25 (located on strand B) and residue Cys80 (located on strand F) [1]. Such disulfide bond has been deemed critical for β_2_m fibrillogenesis at neutral pH [2,3]. Another key structural feature of Hβ_2_m is the existence of a peptidyl-prolyl bond on the BC-loop (between His31 and Pro32), which adopts a thermodynamically unfavorable *cis* isomerization in the native state (Figure 1). Docking of Hβ_2_m onto the β_3_ domain of the MHC involves the four-stranded beta-sheet and a surface area of 595 Å [4]. In particular, the four aromatic residues Phe56, Trp60, Phe62, and Tyr63 and the aliphatic Leu65, which are shielded from the solvent in the quaternary structure of the MHC, become highly solvent-exposed upon dissociation of Hβ_2_m from the heavy chain.

During the continuous turnover of the MHC molecules, Hβ_2_m is shed from the cell membrane and discarded to the serum being catabolized in the kidneys. In individuals with kidney impairment undergoing long-term (>10 years) haemodialysis, the levels of Hβ_2_m increase from 0.3–30 mg/mL to 30–50 mg/mL due to the inability of the dialysis membrane to effectively remove the protein [4]. Clearance failure leads to the onset of a pathological condition termed dialysis-related amyloidosis (DRA). In DRA, Hβ_2_m undergoes extracellular accumulation and slow deposition in the osteoarticular system (tendons, synovia, and bones) where it eventually aggregates into amyloid fibrils. The final outcome of DRA is tissue erosion and destruction.

While a concentration increase of Hβ_2_m appears to be a necessary condition for the occurrence of amyloid formation, it is certainly not sufficient to trigger the amyloid pathway. Indeed, experiments *in vitro* have shown that amyloid fibrils will not form in physiological conditions (of pH and temperature) even when the concentration of Hβ_2_m largely exceeds that observed in DRA patients [5,6]. Although it is widely recognized that WT Hβ_2_m is unable to efficiently nucleate fibrillogenesis *in vitro* under physiological conditions, it is also known that a multitude of environmental factors (e.g., acidic pH [7], agitation [8], addition of Cu^2+^ in the presence of urea [9], co-solvents [10]), can trigger the formation of β_2_m amyloid fibrils in laboratory experiments.

Seminal studies by Chiti and co-workers provided important insights into the *in vitro* folding mechanism of Hβ_2_m in physiological conditions (pH 7.4). Two intermediate states were identified in the folding pathway of Hβ_2_m: an ensemble of partially folded conformers (termed I_1_) with substantial elements of non-random structure that forms from the denatured sate, and a low-populated intermediate state I_2_, forming (directly or indirectly) from I_1_ and presenting a more consolidated protein core [11,12]. Subsequent investigations by Radford and co-workers, which explored the folding mechanism of β_2_m with atomic detail, showed that I_2_ is indeed a highly native-like slow-folding intermediate which is also aggregation-competent at neutral pH and physiological temperature [13]. Despite being native-like, this intermediate exhibits a non-native (*i.e.*, *trans*) isomerization of the peptidyl-prolyl bond between His31 and Pro32, and for this reason, it has been termed I_T_ [13]. It is known that specific variants of Hβ_2_m [13,14], including a cleaved form found in *ex vivo* amyloid fibrils (*i.e.*, ΔN6) [15,16] of patients with DRA, may induce a higher population (up to 90%) of the intermediate I_T_ and efficiently promote β_2_m fibril nucleation and/or elongation in a physiological (or near-physiological) pH in a mechanism akin to conformational conversion in prion [16].

Interestingly, the cleavage of the first six *N*-terminal residues in ΔN6 not only favors the isomerization of the peptidyl-prolyl bond characteristic of I_T_ but also enhances the local dynamics of both the BC- and DE-loops in a pH-dependent manner suggesting these two regions are important for fibril assembly [16]. Indeed, the local dynamics of the region spanning the D-strand and DE-loop, which establishes contacts with the MHC-I heavy chain, has been widely implicated in β_2_m fibrillogenesis [16]. The DE-loop region is a key structural element of the protein that has been shown to be a major determinant of the kinetics of fibril formation being both involved in the nucleation and elongation phases of fibril assembly at acidic pH 2.5 [17]. This segment of the polypeptide chain accommodates several aromatic bulky residues that become solvent exposed in the monomeric (free) form of β_2_m thus being able to act as sticky patches in intermolecular association. Indeed, strand D has been acknowledged to play a major role in edge-edge strand association and fibril assembly [9,18,19]. In this region, residue Trp60 is of particular interest. This bulky amino acid, located at the apex of the DE-loop, is highly conserved amongst vertebrates and plays a critical role in assisting the association of the β_2_m with the MHC-I [20]. These observations have motivated a series of *in vitro* experiments based on mutations of Trp60 and other nearby residues in order to establish their relevance for biological association of β_2_m [20–24].

Here, we use discrete molecular dynamics simulations of a full atomistic Gō model, structural clustering and principal component analysis to assess the impact of two DE-loop mutations (D59P and W60C) on the thermodynamics and folding pathways of Hβ_2_m. While native-centric models have obvious limitations (e.g., they ignore water-mediated effects and cannot be used to explore regions of the free energy landscape dominated by non-native interactions), they are extraordinarily efficient from a computational standpoint, allowing the characterization of biomolecular processes occurring over long timescales such as protein folding. Furthermore, the full atomistic representation adopted in the present work takes into account an essential effect of protein folding which is that of side-chain packing, thus more accurately capturing the effect of single-point mutations [25].

This article is organized as follows. In the next section, we present and discuss the results. In the subsequent section, we provide a brief summary of the model and methods. Finally, we draw the conclusions.

## 2. Results and Discussion

### 2.1. Proteins Studied

In this work, we investigate the equilibrium folding of the (wild-type) WT Hβ_2_m (PDB ID: 1LDS) (Figure 1) and two single-point mutants D59P (PDB ID: 3DHM) and W60C (PDB ID: 3DHJ) obtained by replacing Asp59 by Pro59 and Trp60 by Cys60, respectively. D59P is the more aggregation prone variant studied here, being able to efficiently nucleate fibrillogenesis *in vitro* at physiological pH (7.4) [22]. The W60C mutant, on the other hand, displays a decreased amyloidogenic propensity relatively to the WT form at physiological pH. The amyloidogenic behavior of all variants increases at acidic pH with that of W60C becoming quantitatively comparable to the WT protein [22].

The X-ray structure of the WT Hβ_2_m used in this work displays a straight D-strand with no β-bulge and an outward orientation of the AB-loop [1] which constitutes the most important structural deviations with regard to the conformation adopted by Hβ_2_m in solution and also in the MHC-I [26]. As pointed out in Ref. [27], regular beta-strands are dangerous because they are in the right conformation to interact with other beta-strands they encounter. Thus, this rare conformer of Hβ_2_m is potentially more aggregation prone than other common conformations. Despite being considered a rare conformer of Hβ_2_m, the X-ray structure has been widely used to gain insight into the mechanism of amyloid formation by β_2_m. The X-ray structures of the two mutational variants, W60C and D59P, share with the WT Hβ_2_m X-ray structure its peculiar structural features.

In the current Gō model, the total number of native contacts exhibited by the three variants is 1072 (WT), 1091 (W60C) and 1063 (D59P). It is important to evaluate the impact of each single-point mutation on the number of native contacts established by the DE-loop (which accommodates the mutation), by the (adjacent) BC-loop and also by the terminal regions. The latter are widely acknowledged to have a protective role against aggregation by maintaining the hydrophobic balance that stabilizes the native state [15]. Results are summarized in Table 1.

### 2.2. The Loop Mutations Decrease Thermal Stability

**Figure 2:**
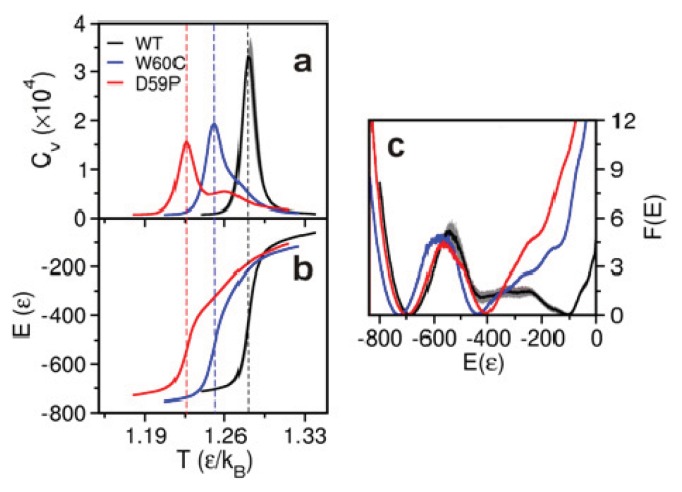
Folding thermodynamics. Temperature dependence of the heat capacity (**a**) and energy *E* (**b**) of the three Hβ_2_m variants and free energy profile along the reaction coordinate *E* at *T_m_* (**c**). The melting (or folding) temperature, *T_m_*, is that corresponding to the peak of the heat capacity curve.

The melting (or transition) temperature *T_m_* is considered a measure of the protein thermal stability. Here, as well as in experiments *in vitro*, we evaluate the melting *T_m_* as the temperature at which the heat capacity *C_v_* peaks (*i.e.*, attains its maximum value). We compute *C_v_* from the mean squared fluctuations in energy at each temperature considered in the RE-DMD simulations, in accordance with the definition *C_v_* = (<*E*^2^> − <*E*>^2^)/*T*^2^. The results for *T_m_* (Figure 2a)—1.280 (WT) > 1.251(W60C) > 1.226 (D59P)—follow the same qualitative trend as those obtained in experiments *in vitro,* 60.1 °C (WT) > 59.8 °C (W60C) > 52.0 °C (D59P) [22], and highlight a clearly more pronounced loss of thermal stability for the D59P mutant. The more pronounced loss of thermal stability exhibited by the D59P variant might be related with its larger propensity to aggregate. Indeed, it forms amyloid fibrils more quickly and with a higher yield than both the WT and W60C mutant [22].

The incipient shoulder in the *C_v_* curve of the W60C mutant at high temperature (*i.e.*, above *T_m_*) indicates a weak pre-folding collapse transition. In the D59P mutant, it is possible to see an incipient second peak in the heat capacity curve (at *T* ~ 1.26) indicating that the pre-folding collapse is a stronger transition in this case. The less sharp folding transition of the D59P mutant is clearly visible in the curve showing the energy variation with temperature (Figure 2b).

### 2.3. The Loop Mutations Trigger the Population of a Partially Folded Intermediate State

**Figure 3:**
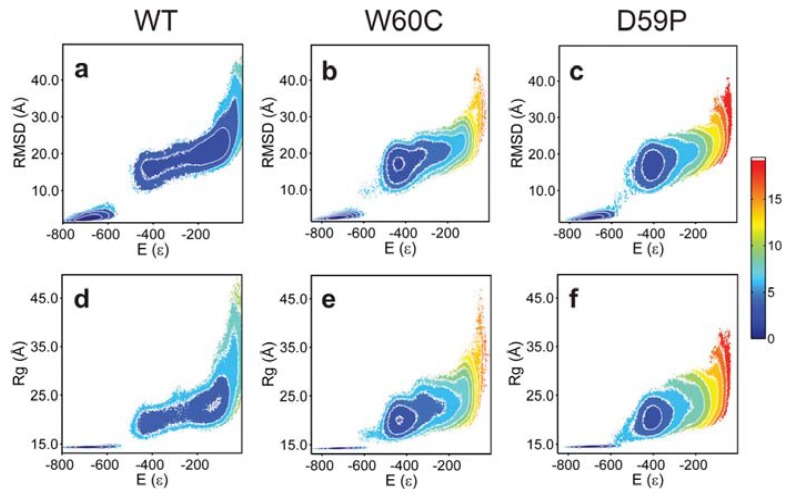
Free energy surfaces. Projection of the free energy on the energy (*E*) and root-mean-square deviation (RMSD) (**a**–**c**) and on the energy (*E*) and radius of gyration (R*_g_*) (**d**–**f**) at *T_m_*.

The analysis of the free energy (FE) profiles (Figure 2c) and FE surfaces (Figure 3) allows for evaluation of the impact of mutations on the folding space of Hβ_2_m. The FE profile for the WT form (Figure 2c) reveals the existence of a free energy barrier between the denatured state and a partially folded intermediate state located at *E*~−400. The free energy barrier is relatively small which indicates that the pre-transition state intermediate forms relatively fast from the denatured state. We therefore hypothesize that it represents the intermediate species I_1_ originally reported by Chiti *et al.* [11,12] or a conformational excursion of the latter that may facilitate its conversion into I_T_. The analysis of the free energy surfaces reveals that the intermediate state is also present in the folding pathways of the mutants sharing the same gross characteristics across the three species: *E* ~ −420, R*_g_* ~ 20 Å and RMSD ~ 15 Å (Figure 3). Interestingly, the TSE of the D59P mutant is slightly stabilized relative to that of the WT Hβ_2_m (Figure 2c) which may facilitate the conformational conversions between the intermediate and the native states. By comparing the free energy profiles of the mutants with that of the WT Hβ_2_m, we observe that the single point mutations thermodynamically stabilize the intermediate state and, most importantly, significantly destabilize the denatured state. Indeed, this effect is so striking that if we had restricted ourselves to the analysis of the free energy profiles of the mutants (Figure 2c) we would have concluded that the free energy minimum located at *E* ~ −400 is not an intermediate state, but a highly native-like denatured state instead (in fact, a compaction and structural consolidation of the denatured ensemble upon loop mutations was reported by Serrano on the Spc-SH3 folding domain [28]). The analysis of the free energy surfaces (in particular the free energy projected onto *E* and RMSD) shows a protrusion of the intermediate state’s basin towards higher energy and RMSD indicating the presence of the denatured state (which, nevertheless, is significantly destabilized at the considered temperature).

The mutations have a minor effect at the level of the native basin. The projection of the FE onto *E* and RMSD shows that the native basin of the WT protein is broader than that of the mutants (Figure 3a–c). Structural clustering (with a cutoff radius of 5–9 Å) of the native ensembles extracted from equilibrated DMD trajectories at fixed temperature (within the transition region) reveal that the WT and, to a lesser extent, the D59P mutant both populate a native-like species exhibiting a partially detached *N*-terminus (Figure S1). The native-like species represents less than 1% of the equilibrium ensemble in both cases.

The partial detachment of the *N*-terminus might reflect a smaller number of native interactions in the termini in the WT and D59P relative to that of W60C (Table 1). On the other hand, in W60C, there is an increment in the number of native contacts (up to 11.4%) established by the first four β-strands relative to the WT form (Table 1), which may contribute to its narrower native basin.

### 2.4. The Intermediate State Has a Molten Globule-Like Nature

**Figure 4:**
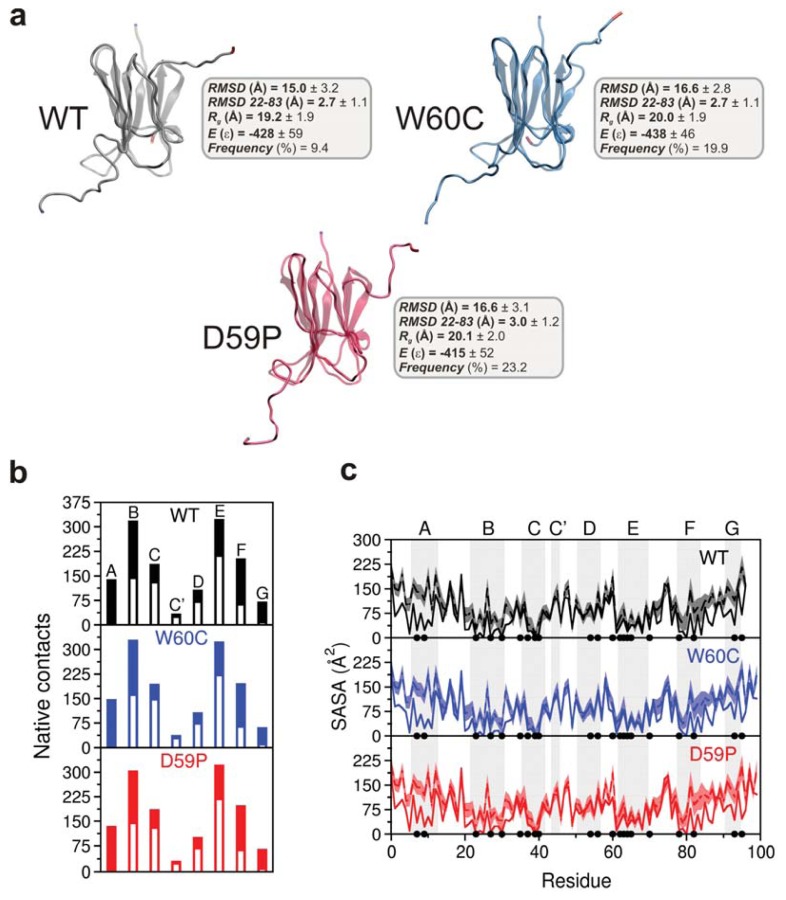
Characterization of the intermediate state identified for each of the three Hβ_2_m variants. (**a**) Three-dimensional structures of the intermediate state fitted to the original X-ray native structure. The (starting) *N*-terminus is colored in blue while the *C*-terminus is shown in red. The intermediate state populates 9.4%, 19.9%, and 23.2% of the equilibrium ensembles of Hβ_2_m (WT), W60C, and D59P, respectively, at their respective *T_m_*; (**b**) Comparison between the average number of native contacts established by each β-strand in the intermediate (white bars) and the number of native contacts by β-strand in the respective PDB native structure; (**c**) Mean SASA per residue in the intermediate state compared with that of the native structure (black line). The shaded area stands for the standard error bars for the mean SASA ensemble average. The black dots represent the 21 hydrophobic core amino acids: Leu7, Val9, Leu23, Val27, Phe30, Ile35, Val37, Leu39, Leu40, Leu54, Phe56, Trp60, Phe62, Tyr63, Leu64, Leu65, Phe70, Tyr78, Val82, Val93, and Trp95.

In order to isolate and structurally characterize the intermediate state populated by each variant, we performed extensive structural clustering (with a cutoff radius of 14–17 Å) over ensembles of conformations collected from equilibrated DMD simulations at fixed temperature within the transition region. We found that the intermediate exhibits the disulfide bond between residues Cys25 and Cys80 (Figure S2), a structural feature which has been proposed to be a pre-requisite for β_2_m fibrillogenesis at neutral pH [3]. The most likely dihedral angle for the His31-Pro32 bond in the intermediate state is ~−90° for the mutants and −45° for the WT. The intermediate’s main structural trait is the existence of unstructured termini detached from the region comprising residues 22–83 (*i.e.*, strands B-F and connecting loops). Given its extension, we term such a region a core region. The RMSD of the core region (<3 Å) calculated with regard to the equivalent region in the native state indicates a high degree of native-like character (Figure S2). This is perhaps associated with the preservation of the disulfide bridge in the intermediate state that secures the integrity of the core region by acting like a staple. The intermediate’s (mean) energy, *E*, represents ~60% of the native energy and its (mean) radius of gyration, R*_g_*, is 35% (WT), up to 40% (W60C, D59P) larger than that of the native state (Figure 4a). The (mean) root-mean-square deviation, RMSD, measured with reference to the native structure ranges from 15 Å (WT) to 16.6 Å (W60C, D59P). These structural traits are consistent with those of a molten globule (MG), *i.e.*, a compact state with fluctuating tertiary structure [29]. The intermediate state populates 9.4% (WT), 19.9% (W60C), and 23.2% (D59P) of the WT, W60C, and D59P equilibrium ensembles, respectively, at *T_m_*.

The comparison between the amounts of native interactions established by each β-strand in the intermediate state with those established in the corresponding native state provides an indication of the loss of native structure (Figure 4b). Apart from losing secondary structure in the terminal strands, the intermediate is also less structurally consolidated in strands B and F. The E-strand establishes the higher number of native contacts thus playing an important role in the preservation of the core region. The loss of native content can be observed with more detail in the probability maps that report the ratios between the total number of native contacts between each pair of residues (computed at the atomic level) in the intermediate and in the native state (Figure S3). The analysis of the probability maps shows that the most ‘promiscuous’ residues (*i.e.*, those involved in a higher number of intramolecular interactions) are located in the E-strand. The similar patterns of native contacts exhibited by the intermediate state of each variant further support the idea that the intermediate state is common to the three protein variants studied here.

### 2.5. The Intermediate State Exhibits Aggregation-Prone Traits

Since the exposure of aggregation-prone hydrophobic patches has been pointed out as a hallmark of protein aggregation [30], we evaluated the solvent accessible surface area (SASA) per residue (Figure 4c) in order to gauge the aggregation potential of the intermediate state. There are similarities in the SASA (per residue) of the intermediate states of all variants, of note, SASA increases from ~3 up to ~40 times with regard to the native state in 38% of the hydrophobic core residues (*i.e.*, Leu7, Val9, Leu23, Val27, Phe30, Leu39, Val82, and Trp95). Apart from the terminal regions (*N*-terminus and strand A plus *C*-terminus and strand G), the increase in SASA is significant for the region comprising B-strand and the BC-loop. These observations thus suggest that the identified intermediate state has a high aggregation potential.

### 2.6. Large Displacements of the Termini Dominate the Intermediate State’s Dynamical Repertoire

Essential dynamics (also known as Principal Component Analysis, PCA) has been traditionally used to identify collective motions in biomolecules. It is a useful method to distinguish the dominant (*i.e.*, essential) motions from the “noise” associated with lower-amplitude motions. High-amplitude loop motions, for example, have been frequently considered to be detrimental for the function of several enzymes. Here, we use PCA to identify the dominant motions of the native and intermediate states in the three variants of Hβ_2_m.

**Figure 5:**
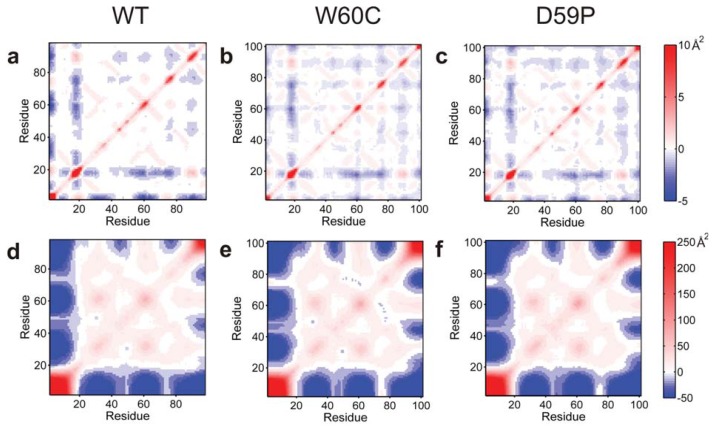
(Cα) covariance matrices (non-mass-weighted) for the native (**a**–**c**) and intermediate states (**d**–**f**). A red point corresponding to a positive covariance value indicates that the two Cα atoms move in a correlated manner (*i.e.*, together) while the blue color indicates that the atoms move into opposite directions. White spots indicate null covariance values, which mean that the Cα movements are not correlated.

We started by calculating the Cα atoms’ variance-covariance matrix for the ensembles of conformations representative of native states that were obtained with the clustering analysis (Figure 5a–c). The native ensembles of the mutants are similar and they exhibit predominantly small-amplitude correlated motions. In the WT, although the pattern of anti-correlated motions is more scattered, they involve a higher-amplitude displacement of the corresponding Cα atoms (Figure 5a). These higher-amplitude motions correspond to the displacement in opposite directions of the *N*-terminus and the BC-, DE-, and FG-loop regions. The AB-loop displays a similar pattern of anti-correlated motion mostly with strands A, B, D and E and both loops DE and EF. In addition there is a clear positive correlation between the movements of the *N*-terminus and the AB-loop. Furthermore, the *N*-terminus moves in tandem with the EF-loop, and the AB-loop does it so with the region enclosing both G-strand and FG-loop. This pattern of positively-correlated motions involving the *N*-terminal regions of the protein is particularly evident for both the WT and, to a lesser extent, for the D59P variants being consistent with the population of the native-like species with a semi-detached *N*-terminus/strand A previously mentioned.

We evaluated the total mean square fluctuations (MSF) of the native state corresponding to the trace of the native covariance matrix. The total MSF of the native state is higher for both the WT and D59P variants (496 and 524 Å^2^, respectively, against 438 Å^2^ for the W60C mutant), which is in agreement with the wider native state basin identified in their FE surfaces. In the WT, the first five eigenvectors or principal components (PCs), which are associated with larger amplitude motions, account for ~54% of the total MSF, while in the mutational variants they account for ~40%

**Figure 6:**
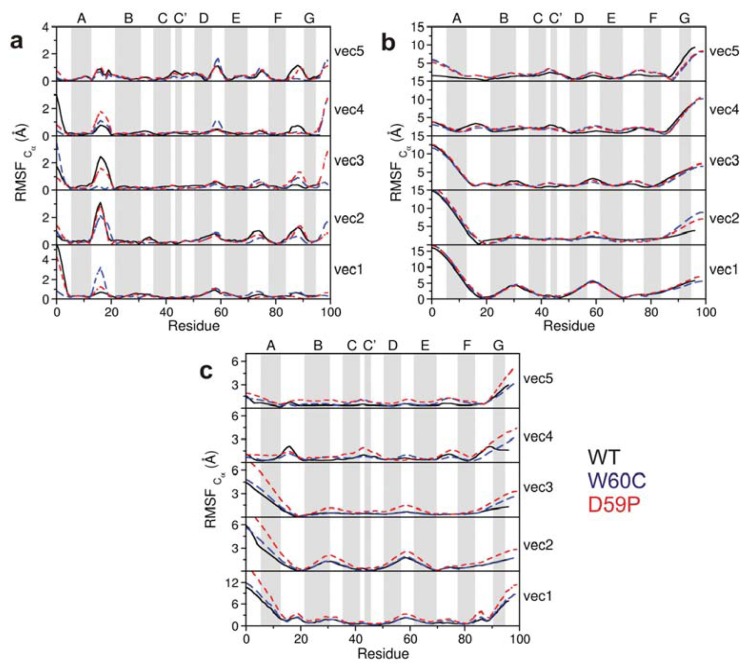
Contribution of each Cα atom to the total RMSF along the first five eigenvectors for the sampled native (**a**), intermediate (**b**), and native and intermediate (**c**) ensembles of the three Hβ_2_m forms.

In order to gain insight into the type of motion associated with the first five PCs, we evaluated the contributions of each Cα atom in these PCs to the total RMSF (Figure 6a). We find that these principal modes represent mostly the movement of both the *N*-terminus and AB-loop suggesting that the transition between the native-state and the native-like state with a semi-detached *N*-terminus occurs mostly along these five principal modes. In the D59P variant along with the motion of the *N*-terminus and AB-loop (PCs 1–3), there is also a high RMSF associated with the *C*-terminus (PCs 3 and 4). Considering only those five eigenvectors, the DE-loop RMSF is only distinctively higher for the W60C variant along PCs 3 and 4. The enhanced dynamics of the native WT and the local nature of the correlated motions which involve well-defined regions of the protein (visible through low sparseness displayed by the covariance matrices) are indicative of highly-directional fluctuations of the native state that might concur for its propensity for *in vitro* native oligomerization at neutral pH [21].

The covariance matrices of the intermediate state’s ensembles of the three variants are quite similar (Figure 5d–f). The *N*-terminal region encompassing the *N*-terminus plus the AB-loop moves in an anti-correlated manner with the BC and DE-loop regions (including corresponding strands) and also with the *C*-terminal region (starting from strand F). In other words, the protein core (plus the *C*-terminal segment) and the *N*-terminal region move along opposite directions. The intermediate state populated by the WT is associated with the largest total MSF (9.4 × 10^4^ Å^2^ against 1.2 × 10^4^, 1.1 × 10^4^ for the D59P and W60C mutants, respectively), which reflects the broader basin observed in its FE surface (Figure 3a). For all three variants the first five eigenvectors (or PCs) account for ~77%–78% of the total MSF in the considered DMD trajectories at *T_m_*. The contributions of each C*α* atom in these PCs to the total RMSF (Figure 6b) show very similar behavior across the three protein variants, and highlight especially high RMSF associated the motion of the *N*- and *C*-terminal regions. These motions have much larger amplitudes than the equivalent motions in the native state.

The first five principal modes of the combined covariance matrices (Figure 6c) highlight the enhanced RMSF associated with the motion of the *N*- and *C*-terminal regions of the D59P mutant responsible for the transition from the native to the intermediate state. The *N*-terminal region should be the first to become detached from the protein core (along PC 1 to 3).

### 2.7. Is the Predicted Intermediate State Compatible with a Molten Globule Identified for Hβ_2_m at pH 4?

The importance of a MG state for Hβ_2_m *in vitro* fibrillogenesis was reported for pH = 3.6 [31]. This MG state appears to be preferentially stabilized at pH ~ 4 [32] reaching a maximum (equilibrium) population of 76% in those conditions [33]. It was shown to be the precursor of short, curved, worm-like fibrils [34]. Structurally speaking, it preserves the disulfide bond Cys25–Cys80, it exhibits a stable and compact core involving strands B, C, D, E and F, and highly unstructured terminal strands A and G [7,31–35]. These structural traits are—all of them—shared with the intermediate state reported in the present work (Figure 4b). Despite these remarkable structural similarities, we cannot conclude that the intermediate reported here would form and be thermodynamically stable at pH 4.0 because its prediction is based on a Gō modeling approach that does not take into account the effect of pH. However, in what follows, we present an argument that suggests that the population of our intermediate state may be favored at pH 4.0, leading to a higher thermodynamic stability at this pH. Therefore, we cannot exclude the possibility that the MG state detected *in vitro* and the intermediate state reported here represent the same species.

As the pH decreases from 7.0 to 4.0, the number of positive charges in the native states of the WT and mutant forms increases (Table S1). In particular, according to PROPKA [36] predictions, the native state’s *N*-terminal region (extending up to residue 21) acquires one additional positive charge, and loses one negative charge, while the *C*-terminal region (starting at His84), displays one additional positive charge. The increased load of positive charge at pH 4.0 is visible in the electrostatic isocontours of the native and intermediate conformations (Figure S4). In particular, the excess of positive charges in both termini in the native structure is likely to drive their separation, contributing to the formation of the intermediate state at pH 4.0. However, the detachment of both protein termini from the core exposes hydrophobic residues that account for an unfavorable change in the nonpolar free energy of solvation (*G*_solv,nonpolar_) (Table S1). Still, solvation calculations suggest that formation of the intermediate state is more likely to occur at pH 4 because at this pH the electrostatic contribution to the free energy of solvation (*G*_solv,polar_) in the intermediate state is considerably lower than at pH 7.0 (Table S1).

## 3. Experimental Section

In this section we provide a brief description of the model and methodologies employed in this work. Detailed explanations can be found elsewhere [25,37,38].

### 3.1. Gō Model and Discrete Molecular Dynamics Simulations

We consider a representation of the protein where all non-hydrogen atoms are represented by hard spheres of unit mass. The size of each atom is defined by scaling the relevant van der Waals (vdW) radius [39] by a factor α < 1. Protein energetics contains excluded volume interactions, non-bonded interactions and bonded interactions, all of which are modeled by discontinuous, piecewise constant interaction potentials. To take into account excluded volume interactions, all atom-atom interactions at distances smaller than the sum of the hard-sphere radii (hard clashes) are strictly forbidden. A pair of atoms *i* and *j* separated by a distance *r_ij_* is a hard clash if *r_ij_* < α(*r_0i_* + *r_0j_*), where *r_0i_* is the vdW radius of atom *i*. Non-bonded (or contact) interactions are represented by a square-well potential whose depth is given by Gō energetics [40]. This means that if atoms *i* and *j* are located in residues which are separated by more than two units of backbone distance, the interaction parameter between them, *ɛ_ij_*, is given by:

(1) $${ɛ}_{ij}=\{\begin{array}{lll} \infty & \text{if} & r_{ij}<\sigma \\ \Delta_{ij} & \text{if} & \sigma \leq r_{ij}<\lambda \sigma \\ 0 & \text{if} & r_{ij}\geq \lambda \sigma \end{array}$$

In the expression above, σ =α (*r_0i_+ r_0j_*) is the hard-core distance, λ is a scaling factor that controls the range of attractive interactions, and Δ*_ij_* = −1 if *i* and *j* are in contact in the native conformation and is 0 otherwise. We followed [41] in treating the energetics of the disulfide bond in the same manner as we treat the other contact interactions. We set α = 0.80 and λ = 1.6 in order to have a well-behaved folding transition [38,41]. This choice of parameters sets a cut-off distance of 4.7 Å (for methyl carbon), and leads to 1072, 1091, and 1063 native contacts in the WT Hβ_2_m (PDB ID: 1LDS), W60C (PDB ID: 3DHJ) and D59P forms (PDB ID: 3DHM). The total energy of a conformation is computed as the sum over all atom pairs,

(2) $$E=\sum_{all\;pairs}{ɛ}_{ij}$$

Covalent and covalent-like bonds between adjacent atoms *i* and *j* are modeled by a narrow, infinitely high potential well,

(3) $$u_{i,j}=\{\begin{array}{lll} 0 & \text{if} & b_{0}<\;|r_{\text{i}}-r_{j}|<b_{1}\\ \infty & \text{if} & |r_{\text{i}}-r_{j}|\;\leq b_{0}\;\text{or }|r_{\text{i}}-r_{j}|\;\geq b_{1}, \end{array}$$

with *b*_0_*=* 0.9 and *b*_1_*=* 1.1, so that the average covalent bond length is equal to 1 Å. Folding dynamics is modeled with discrete (also termed discontinuous) molecular dynamics simulations [42,43]. The evaluation of thermodynamic properties requires equilibrium sampling of the conformational space. In order to obtain reversible folding trajectories at different temperatures, we used replica-exchange (RE) Monte Carlo simulations [44]. In order to compute thermodynamic properties (e.g., the heat capacity), we used thermally equilibrated and uncorrelated states from very long DMD trajectories (consisting of at least 40 billion events after thermal equilibration). The weighted histogram analysis method (WHAM) [45] was used to compute the free energy as a function of different reaction coordinates (energy, *E*, radius of gyration, *R*_g_, and root-mean-square deviation*,* RMSD).

### 3.2. Structural Clustering

Apart from the DMD-RE simulations, we also ran extensively long (up to 4 × 10^11^ events) DMD simulations at fixed temperature *T* (with *T* located within the transition region of each variant). A total number of 3 (D59P) and 6 trajectories (W60C) were considered. From each set of simulations we constructed a conformational ensemble for each analyzed protein (with 1.2–2 × 10^5^ elements) by picking up equilibrated and uncorrelated conformations (*i.e.* conformations recorded every 50,000 events beyond the first folding transition) from the DMD trajectories. Subsequently, each conformational ensemble was analyzed with the *k*-means clustering algorithm of Brooks and co-workers as implemented in the MMTSB toolset [46]. Data recorded at fixed temperature was also used for principal component analysis (PCA).

### 3.3. Principal Component Analysis

The PCA method is commonly used to identify the so-called essential motions, *i.e.*, the set of collective internal fluctuations that make up the most important contributions to protein dynamics on a large number of conformations taken from a molecular dynamics trajectory. The PCA analysis presented in the present work was carried out with the g_covar and g_anaeig modules of GROMACS 4.5.4 [47]. The variance-covariance matrices were evaluated after optimally superimposing the analyzed conformations onto the respective X-ray native structure. Further details can be found in [25].

## 4. Conclusions

In this work, we used molecular dynamics simulations to explore the impact of two selected loop mutations (D59P and W60C) on the folding pathways of a rare conformer of Hβ_2_m lacking a beta-bulge in strand D. Despite its peculiar features, this conformation has been widely used to study Hβ_2_m aggregation. In particular, the mutant variants considered here were prepared by amino acid replacement using this conformation as a template. Experiments *in vitro* indicate that the D59P mutational variant is more amyloidogenic than the WT protein, and the W60C is less. At acidic pH (2.5), the WT and W60C forms exhibit similar aggregation efficiencies.

The simulation results reported here recapitulate the trend for the melting temperature observed in experiments *in vitro*. Namely, that the largest decrease in *T_m_*, reflecting the most significant loss of thermal stability, occurs for the more aggregation prone mutant D59P. Furthermore, our structure-based model predicts that both mutations induce the population of a partially folded state with 60% of the native’s state enthalpy, which has some molten globule-like character. The population of this folding intermediate is larger for the more amyloidegenic variant. The structural characterization of the intermediate state shows that about 40% of its hydrophobic core residues become significantly solvent exposed. The exposition of hydrophobic patches is a classical hallmark of protein aggregation. In addition, the folding intermediate identified here also presents two unstructured termini. The relevance of this particular structural feature for protein aggregation has been recently acknowledged [37,48,49]. In fact, perturbation of the *N*- and *C*-terminal strands has been demonstrated to account for the generation of assembly-competent states of β_2_m [14–16,50]. Furthermore, an unstructured strand A has been previously linked with the onset of β_2_m fibrillogenesis given its involvement in one of the two types of dimer interfaces established between monomers within the β_2_m H13F hexamer [9]. Thus, it is likely that the folding intermediate identified in this study is also an intermediate state for aggregation. In addition, it is also possible that it represents a structurally consolidated version of the intermediate I_1_ that facilitates its conformational transition into I_T_, a widely recognized amyloid competent species in the folding space of β_2_m.

The W60C mutant also populates the intermediate state identified here. However, the W60C mutant is less amyloidogenic than the WT despite exhibiting a larger intermediate population. This may be taken as an indirect indication that the large and bulky tryptophan 60 is an important hub for the establishment of intermolecular interactions. The importance of Trp60 in β_2_m aggregation has been widely acknowledged [9,19–22].

The principal component analysis carried out in this study showed that the principal modes of the intermediate state are associated with the termini. In particular, the *N*-terminus moves significantly and in an opposite direction from the rest of the protein. It is well known that high-amplitude motions become overdamped in the presence of water, which is the major source of friction for slow and especially, very slow modes [51]. The fact that the high amplitude motions will be overdamped in water does not change one of the hypothesis that we put forward in our work, *i.e.*, that the identified intermediate state is aggregation-prone. Indeed, the major driving force for aggregation is the exposition of hydrophobic core residues, and in the present study, this trait comes in association with the loss of secondary structure in the protein termini and not with their mobility. However, one can conjecture that a more mobile terminus is more likely to establish intermolecular interactions. In this regard, overdamping the termini movement may have a minor effect on the predicted aggregation potential of the identified intermediate state, especially in the case of the D59P mutant, for which these movements are particularly relevant.

The population of a molten globule state has been reported for several proteins [52,53], including β_2_m [7,31–35]. The molten globule state detected for β_2_m shares with the intermediate state identified here important structural features. Namely, the preservation of the disulfide bond Cys25–Cys80, a stable and compact core, and highly unstructured termini. It has been suggested that the protonation of His84 (which will happen at acid pH given its low p*K*_a_) is a key event that triggers the molten globule formation [32]. We put forward a simple argument that suggests that pH 4 may favor the population of the intermediate state reported here. Thus, we cannot exclude the possibility that the intermediate state reported here and the *in vitro* detected molten globule state correspond to the same species. Since the intermediate state reported here displays aggregation-prone traits, the higher stabilization of the intermediate state at pH 4 may provide a rationale for the enhanced amyloidogenic potential of all the protein sequences studied here at acidic pH.

## Supplementary Information

Figure S1 Three-dimensional structures of the scarcely-populated native-like species identified for two of the Hβ_2_m variants fitted to the original X-ray native structure. The (starting) *N*-terminus is colored in blue while the *C*-terminus is shown in red.

Figure S2 Additional structural traits of the intermediate state identified for the three Hβ_2_m variants. The graph displays on the left-hand side the distributions of Cα RMSD values of the core region, comprehended between β-strands B and F (residues 22–83), after fitting it to the corresponding native structure core. The right-hand side depicts the distributions of disulfide bridge bond-length values in the three variants.

Figure S3 Native contact maps of the intermediate state identified for each of the three Hβ_2_m variants. Bottom half: Native contacts by residue in the native state. Top half: Probability map of the ratios between the total number of native atomic contacts by pair of residues in the intermediate and native states.

Figure S4 Electrostatic isocontours of the WT native (N)—X-ray structure—and intermediate (I)—clustering representative—states in acidic (4.0) and neutral pH (7.0) conditions. Coloring from red to blue corresponds to electrostatic potentials of −5 to +5 k_B_T. The 21 hydrophobic core amino acids are represented in pink in the SASA representation/depiction of the protein at the bottom of the figure. The protonation states at both pH values were attributed with PROPKA [36] via the webserver PDB2PQR v1.8 [54]. The Poisson-Boltzmann equation was solved using the Adaptive Poisson-Boltzmann Solver APBS v1.4 [55] in VMD v1.8.7 [56]. All isosurfaces were generated at −5 and +5 k_B_T.

**Table S1:** Electrostatic and nonpolar contributions (in kJ mol^−1^) to the free energy of solvation of the native (N) and intermediate (I) states of each Hβ_2_m form at pH 4.0 and 7.0. The total net charge of each species at both pH values is registered between parentheses. The native state calculations used the original X-ray structures while the intermediate estimates considered one clustering representative of each variant. In order to obtain estimates of the free energies of solvation of both the native and molten globule states of the three Hβ_2_m forms at pH 4.0 and 7.0 we calculated both the electrostatic/polar and nonpolar contributions to the these free energies. The electrostatic contribution to the free energy of solvation, *G*_polar,solv_, was obtained through the solution of the Poisson-Boltzmann equation which switches on an electrostatics continuum. This calculation was performed with the APBS software package [55]. The interior dielectric constant was set to 2 while the dielectric constant of water was set to 78.54. The atomic charges and radii used in the PB calculations were extracted from the AMBER ff99 force field (in the PDB2PQR software [54]). The nonpolar contribution to the free energy of solvation, which accounts for the burial of the solvent-accessible surface area (SASA) upon binding, was obtained as *G*_nonpolar,solv_ = γ × (SASA) + β. The solvent-accessible surface area was calculated with GROMACS v4.5.4 with a 1.4 Å radius probe. The parameters γ and β were set to 2.2 kJ mol^−1^ nm^−2^ and 3.8 kJ mol^−1^, respectively.

Protein	pH	*G* _solv,polar_ (150 mM NaCl)	*G* _solv,nonpolar_
WT N	4 (8e)	2841	146
	7 (−2e)	2991	

WT I	4 (11e)	3263	
	7 (−3e)	3528	205

W60C N	4 (11e)	3029	151
	7 (−2e)	3328	

W60C I	4 (11e)	3676	222
	7 (−2e)	3990	

D59P N	4 (9e)	3075	156
	7 (−1e)	3347	

D59P I	4 (9e)	3769	217
	7 (−1e)	4112	

## Figures and Tables

**Table 1:** Native contacts established by β-strand and loop. The change (in percentage) in the number of native contacts relatively to the WT is shown in parentheses. The WT form and the D59P mutational variant display a similar number of native contacts in the DE-loop (44 and 46, respectively) in agreement with the similar conformations adopted by the DE-loop in both cases. The two additional native contacts observed in the D59P mutant may result from an increased rigidity brought by the proline side-chain. The Trp60 to Cys single-point mutation in the W60C form produces the most noticeable impact on the number of native contacts established by the DE-loop which decreases by −13.6% relatively to the WT. This observation reflects the substitution of Trp60 residue, a potential hub for intra-molecular interactions due to its large size and bulkiness. This decrease in the number of native contacts extends to the adjacent BC-loop. There is a loss of native contacts in the termini of the D59P variant relatively to the WT (3% in the *N*-terminus and 10% in the *C*-terminus). In the W60C variant, the loss of native contacts in the *C*-terminus increases up to 12.5%. In the case of the amyloidogenic variant (D59P), there is a noticeable decrease in the number of contacts established by the loops connecting the terminal strands. In the case of the less-amyloidogenic variant W60C, the number of contacts established by the terminal loops is only slightly lower than the ones displayed by the WT.

β-strand	WT	W60C	D59P
A (6–12)	140	149 (+6.4%)	136 (−2.8%)
B (22–30)	319	330 (+3.4%)	305 (−4.4%)
C (36–41)	187	195 (+4.3%)	187
C′ (44–45)	35	39 (+11.4%)	32 (−8.6%)
D (51–56)	108	108	102 (−5.6%)
E (62–69)	324	325 (+0.3%)	323 (−0.3%)
F (78–83)	204	197 (−3.4%)	199 (−2.4%)
G (91–94)	72	63 (−12.5%)	66 (−8.3%)

**Loop**	**WT**	**W60C**	**D59P**

AB (13–21)	89	87 (−2.2%)	80 (−10.1%)
BC (31–35)	102	92 (−9.8%)	97 (−4.9%)
DE (57–61)	44	38 (−13.6%)	46 (+4.5%)
EF (70–77)	111	100 (−9.9%)	108 (−2.7%)
FG (84–90)	95	94 (−1.0%)	88 (−7.4%)
