# Supplementary figures and images for: Assessing the Effect of Loop Mutations in the Folding Space of β2-Microglobulin with Molecular Dynamics Simulations
Source: Int J Mol Sci. 2013 Aug 22;14(9):17256–78. doi: 10.3390/ijms140917256 (PMC3794727; doi:10.3390/ijms140917256)

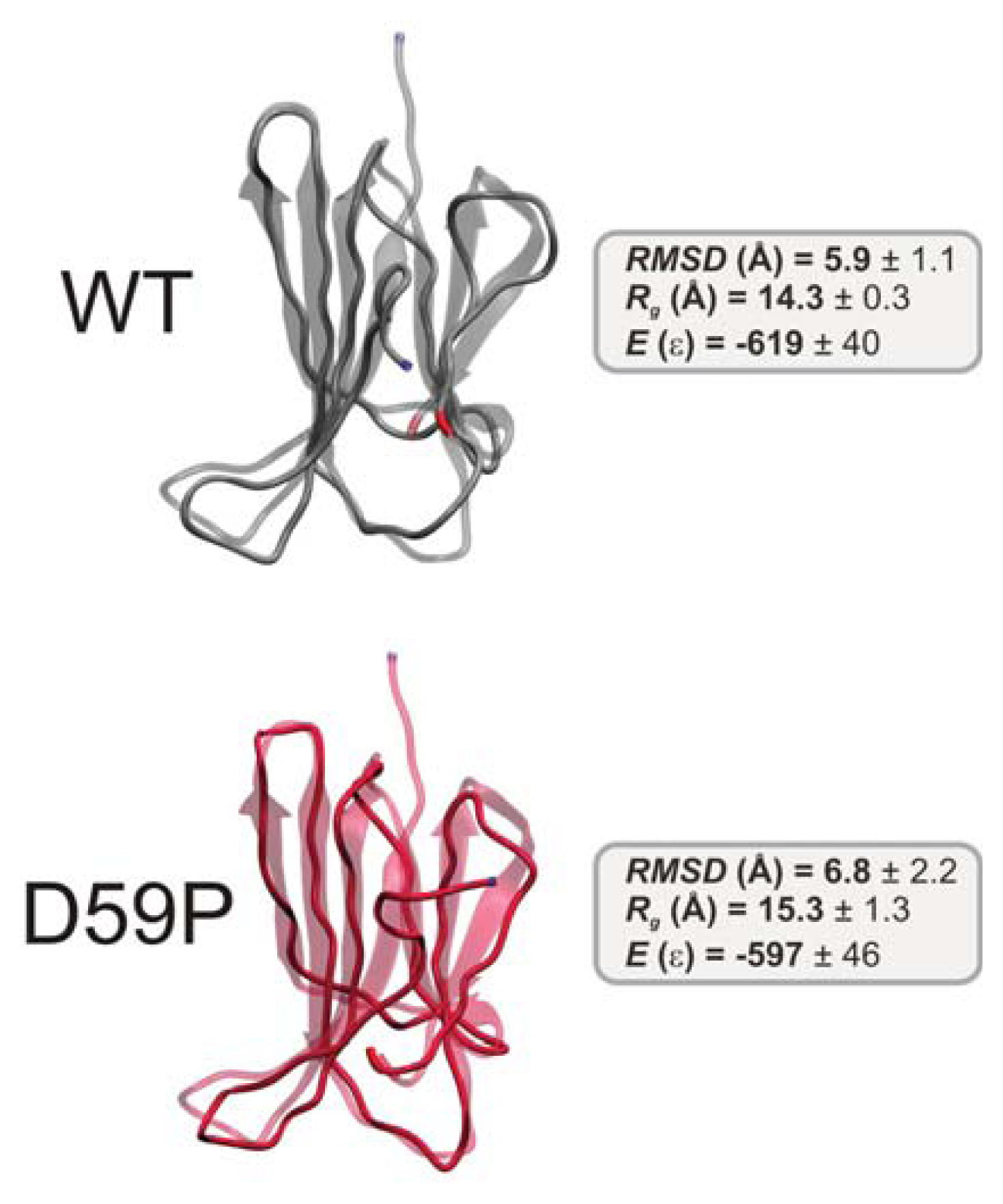

Supplement: Figure S1 — Three-dimensional structures of the scarcely-populated native-like species identified for two of the Hβ2m variants fitted to the original X-ray native structure. The (starting) N-terminus is colored in blue while the C-terminus is shown in red. [file ijms-14-17256s1.tif]

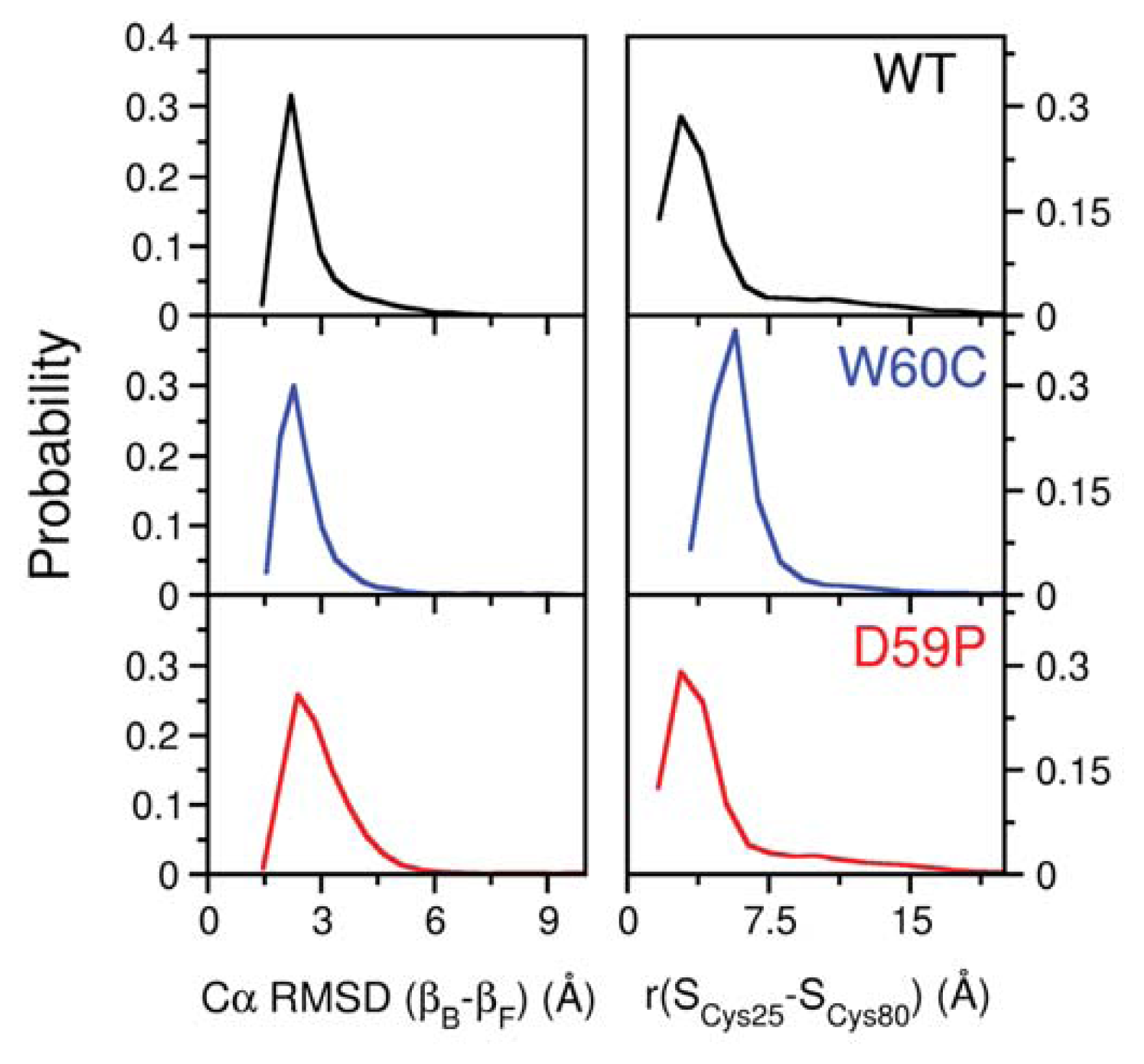

Supplement: Figure S2 — Additional structural traits of the intermediate state identified for the three Hβ2m variants. The graph displays on the left-hand side the distributions of Cα RMSD values of the core region, comprehended between β-strands B and F (residues 22–83), after fitting it to the corresponding native structure core. The right-hand side depicts the distributions of disulfide bridge bond-length values in the three variants. [file ijms-14-17256s2.tif]

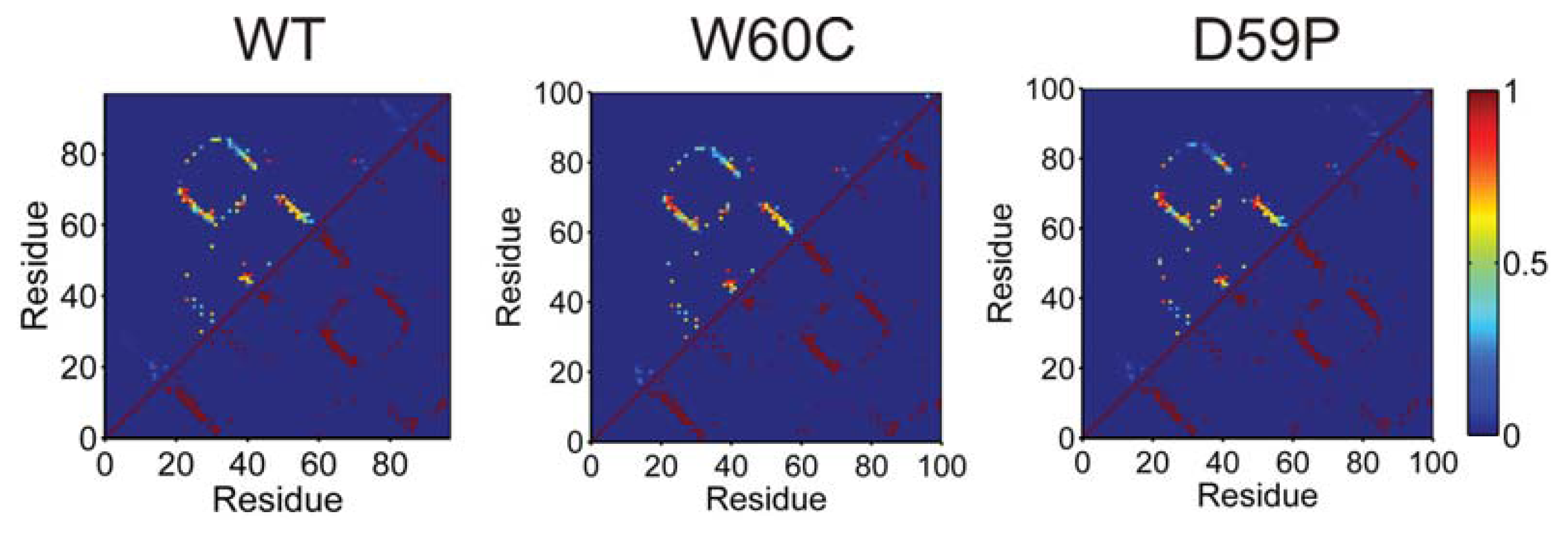

Supplement: Figure S3 — Native contact maps of the intermediate state identified for each of the three Hβ2m variants. Bottom half: Native contacts by residue in the native state. Top half: Probability map of the ratios between the total number of native atomic contacts by pair of residues in the intermediate and native states. [file ijms-14-17256s3.tif]

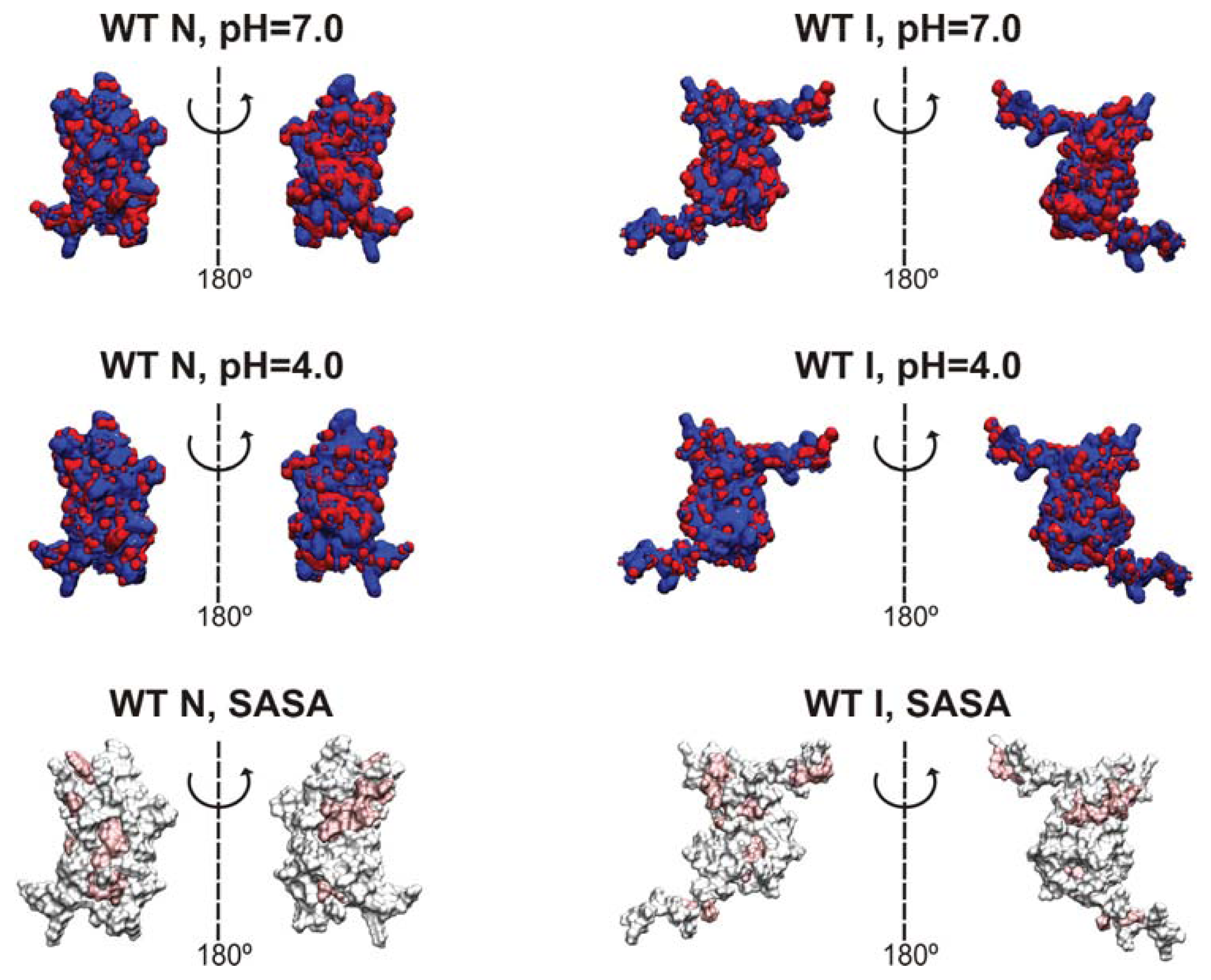

Supplement: Figure S4 — Electrostatic isocontours of the WT native (N)—X-ray structure—and intermediate (I)—clustering representative—states in acidic (4.0) and neutral pH (7.0) conditions. Coloring from red to blue corresponds to electrostatic potentials of −5 to +5 kBT. The 21 hydrophobic core amino acids are represented in pink in the SASA representation/depiction of the protein at the bottom of the figure. The protonation states at both pH values were attributed with PROPKA [36] via the webserver PDB2PQR v1.8 [54]. The Poisson-Boltzmann equation was solved using the Adaptive Poisson-Boltzmann Solver APBS v1.4 [55] in VMD v1.8.7 [56]. All isosurfaces were generated at −5 and +5 kBT. [file ijms-14-17256s4.tif]
